# Advancing the understanding and management of angioimmunoblastic T-cell lymphoma: insights into its pathogenesis, clinical features, and emerging therapeutic strategies

**DOI:** 10.3389/fonc.2025.1479179

**Published:** 2025-03-03

**Authors:** Yurou Xing, Junmeng Huang, Yi Zhang, Yongsheng Wang, Shaochong Qi

**Affiliations:** ^1^ Thoracic Oncology Ward, Cancer Center, West China Hospital, Sichuan University, Chengdu, Sichuan, China; ^2^ Department of Neurology, The First Affiliated Hospital of Chongqing Medical University, Chongqing, China; ^3^ The Quzhou Affiliated Hospital of Wenzhou Medical University, Quzhou People’s Hospital, Quzhou, China

**Keywords:** angioimmunoblastic T-cell lymphoma (AITL), mutations, immunophenotypic, treatment, prognostic

## Abstract

Angioimmunoblastic T-cell lymphoma (AITL) is a clinically aggressive non-Hodgkin lymphoma associated with many immune disorders. The incidence of AITL has gradually increased in Asia in recent years. Malignant AITL cells originate from T follicular helper cells, which have a unique morphology and complex biological characteristics. High-throughput sequencing studies have identified many gene mutations associated with its pathogenesis, including mutations in tet methylcytosine dioxygenase 2 (TET2), isocitrate dehydrogenase (NADP+) 2 (IDH2), DNA methyltransferase 3 alpha (DNMT3A), ras homolog family member A (RHOA), and T cell receptor-related genes. Currently, there is no standardized treatment for AITL, the first-line chemotherapy is ineffective, the recurrence rate is high, the overall prognosis of patients is poor, and the median survival time does not exceed three years. New drugs are urgently needed. However, with continuous in-depth study of the molecular genetic mechanism of AITL, some new drugs and therapies have been tested for patients with relapsed and refractory AITL, achieving some therapeutic effects. Increasing clinical studies are evaluating new potential targets for AITL based on specific molecular markers, gradually improving individualized treatment and ultimately improving the clinical prognosis of patients with AITL. This review first summarizes the progress of research on the etiology, clinical pathological characteristics, and molecular genetic mechanisms of AITL to enhance understanding of the disease. It then summarizes the progress of research on its treatment strategies to provide some references for clinically diagnosing and treating AITL.

## 1 Introduction

Angioimmunoblastic T-cell lymphoma (AITL) represents a prevalent subtype of peripheral T-cell lymphoma (PTCL), comprising 15% to 20% of all PTCL cases (1). AITL arises from T follicular helper (TFH) cells and primarily occurs in lymph nodes (2). AITL is more common in older men. Most patients are in the late stage when they are first diagnosed. Its main clinical manifestations are lymphadenopathy and B symptoms unique to lymphoma (fever, night sweats, weight loss), which may also be accompanied by hemolysis, anemia, hepatomegaly, and splenomegaly. TFH cells interact with B cells to promote the proliferation, differentiation and antibody production of B cells in physiological condition. Abnormalities in TFH cells in AITL patients can lead to B-cell dysfunction, which can trigger a range of symptoms of autoimmune disorders. The diagnosis of AITL may be delayed because the immune symptoms are not obvious or resemble those of other diseases. Laboratory tests can show abnormal results such as increased lactate dehydrogenase (LDH), reactive plasma cells, and polyclonal globulin. Up to 70% of patients with AITL may have bone marrow involvement, manifested as polymorphic cell infiltration around trabeculae and in the interstitium (3). More than 50% of patients with AITL also have skin symptoms, such as maculopapular rash and itching (4).

The pathogenesis of AITL has not yet been fully elucidated. Its onset is associated with mutations in genes such as tet methylcytosine dioxygenase 2 (*TET2*), isocitrate dehydrogenase (NADP^+^) 2 (*IDH2*), DNA methyltransferase 3 alpha (*DNMT3A*), ras homolog family member A (*RHOA*), and T-cell receptors (TCRs). There is currently no unified treatment for AITL. The standard first-line treatment still adheres to the CHOP regimen (cyclophosphamide, doxorubicin, vincristine, and prednisone) or a CHOP-like regimen. However, the efficacy is poor, the remission period is short, the recurrence rate is high, and overall survival (OS) and failure-free survival are low (5). However, with an increasing understanding of its pathogenesis in recent years, various new treatment regimens and drugs have emerged, and new immune molecular drug treatments have also achieved good results (6).

This review summarizes the clinical pathological characteristics of AITL, research progress on its molecular genetic mechanism, progress on its treatment strategies, and factors related to poor prognosis to provide some reference for clinically diagnosing and treating AITL.

## 2 Pathological characteristics

AITL displays unique pathological features crucial for its diagnosis. Histologically, it involves partial or complete disruption of lymph node architecture, significant proliferation of blood vessels, and expansion of follicular dendritic cell networks. A polymorphous infiltrate of neoplastic T cells and various inflammatory cells creates a complex background that can obscure diagnosis. Immunophenotypically, the neoplastic cells express T follicular helper (TFH) cell markers such as CD4, PD-1, and CXCL13, confirming their origin. Understanding these pathological characteristics is essential for accurate diagnosis and has important implications for targeted therapy.

### 2.1 Main histological characteristics

The lymph node structure is destroyed or partially or wholly disappeared, the germinal center is missing, and, in some cases, only the remaining follicular structure is visible. In the paracortical area, there was a marked proliferation of dendritic hyperendothelial veins (HEVs), which were often branched. HEVs serve as conduits for lymphocytes from the blood to lymphoid organs and lymphoid tissues. It is essential for the node-initiated immune response (7). Eosinophilic material deposition can be seen in the interstitium and blood vessel walls. Follicular dendritic cells proliferate to form a network structure partially located around the proliferating small blood vessels. Tumor cells have abnormal morphology and are small to medium in size, with clear or lightly stained cytoplasm, oval or round nuclei, and fine dust-like chromatin. Tumor cells often form clusters and proliferate around blood vessels. B immunoblast-like large cells can be scattered in the paracortical area, some of which are Reed-Sternberg cell-like, with a B-cell phenotype, and are positive for the Epstein–Barr (EB) virus. In the background, many inflammatory cells, such as plasma cells, histiocytes, epithelial cells, eosinophils, and immunoblasts, can be seen infiltrating the tumor, with tumor cells only accounting for a minority of the tissue composition. This infiltration is because AITL originates from TFH cells, which can release various cytokines and chemokines, recruit various types of inflammatory cells, and infiltrate in large numbers, forming a complex inflammatory background for AITL (8). This complex background cellular component is also one of the main reasons why AITL is easily misdiagnosed.

AITL has a pathomorphological evolution. In pattern 1, the architecture of the lymph nodes is well preserved and the lymphoid follicles are hyperplastic, but often lack a clear mantle area and poorly demarcated cortical boundaries. Similar to reactive hyperplasia of lymph nodes, this pattern is difficult to recognize and prone to misdiagnosis. In pattern 2, most of the lymph node architecture was destroyed and the remaining follicles showed degenerative changes. In pattern 3, the lymph node architecture is completely destroyed and replaced by tumor tissue, and no follicles are present. Follicular dendritic cells proliferated irregularly with extensive vascular proliferation and pleomorphic infiltration. This is usually typical of the disease (9).These growth patterns reflect the histological features and pathological changes of AITL at different stages.

### 2.2 Immunophenotypic characteristics

AITL tumor cells can characteristically express various immune phenotypes on their surface, including pan-T cell differentiation antigens such as CD2, CD3, CD4, CD5, and CD8. Gene expression profile analysis showed that Global gene expression patterns seen in whole AITL tissues and isolated tumor cells were then shown to resemble those seen in TFH cells. The molecular characteristics of AITL were similar to the expression pattern of TFH cells, including the expression of CXCL13, BCL6, PDCD1, CD40L, CD200 and other TFH-related genes. The results strongly support that TFH cells are the normal counterpart of AITL. These TFH surface markers are the most commonly used and sensitive markers for confirming the AITL diagnosis. It facilitates better diagnosis of the disease and provides a basis for understanding the pathogenesis of AITL. At the same time, new therapeutic strategies can be developed based on the molecular markers and signaling pathways unique to TSH cells, which helps to identify therapeutic targets and make treatment more targeted (7, 10). TNF receptor superfamily member 8 (TNFRSF8/CD30) is sometimes also expressed, which has become a new direction for immune-targeted therapy. The expression of complement C3d receptor 2 (C2/CD21) and Fc epsilon receptor II (FCER2/CD23) can specifically show the irregular proliferation of follicular dendritic cells. In addition, CD84 and interleukin 21 (IL21) are also closely related to TFH cells.

## 3 Genetic and molecular mechanism studies

AITL exhibits chromosomal gains in chromosomes 3, 5, 11q13, 18, 19, and 22q. Over 75% of patients have monoclonal T-cell receptor (TCR) gene rearrangements, and 25%–30% have immunoglobulin clonal gene rearrangements. Common mutations occur in genes such as RHOA (11), TET2 (12), DNMT3A (13), IDH2 (14), and TCR pathway-related genes like PLCG1 (15), CD28 (16), and FYN (17).

### 3.1 RHOA G17V mutation

RHOA encodes a small GTPase involved in cell processes like proliferation and survival. Mutations in RHOA are present in 50%–70% of AITL cases, with over 90% being the G17V mutation (18). This mutation activates the TCR pathway by binding to VAV1 (19), potentially contributing to AITL development. Mouse models with the RHOA G17V mutation exhibit AITL-like lesions, underscoring its carcinogenic potential (20). RHOA mutations often coexist with TET2 mutations, suggesting a synergistic effect on TFH cell differentiation and AITL induction (11).

### 3.2 Epigenetic regulator-related mutations

*TET2 mutations:* TET2-inactivating mutations occur in 70%–80% of AITL cases and are considered early events in its development (12). They affect hematopoietic stem and progenitor cells by promoting self-renewal and differentiation into specific myeloid lineages (21). TET2 mutations can lead to hypermethylation of the BCL6 gene’s first intron, upregulating BCL6 and promoting the growth of TFH-like cells (22). TET2 mutations often coexist with RHOA mutations, with studies confirming their synergistic effects in mouse models (23).

*DNMT3A mutations:* DNMT3A encodes a methyltransferase involved in DNA methylation. Mutations reduce its activity and are found in 10%–25% of AITL cases, often alongside TET2 mutations (13). These mutations synergistically promote hematopoietic stem cell self-renewal and malignant transformation (24). Mouse models show that co-mutations of TET2 and DNMT3A increase DNA methylation of tumor suppressor genes and activate the oncogenic Notch pathway (25).

*IDH2 mutations:* IDH2 mutations are present in 20%–30% of AITL cases and are specific to position R172 (14). These mutations produce 2-hydroxyglutarate, affecting DNA and histone demethylation and promoting lymphoma development. IDH2 mutations often coexist with TET2 mutations, enhancing TFH-related gene expression and histopathological features like angiogenesis (26).

### 3.3 TCR Pathway-related mutations

Recurrent genetic changes in TCR pathway genes such as PLCG1 (15), CD28 (16), VAV1, and FYN (17) occur in nearly half of AITL cases. CD28-CTLA4 fusion mutations exist in about 60% of cases, potentially converting inhibitory signals into stimulatory ones and continuously activating T cells (27). FYN activating mutations disrupt inhibitory interactions and enhance tyrosine kinase activity (28). Fusion of FYN with TRAF3IP2 leads to abnormal NF-κB signaling and T-cell transformation (29). Mutations in CARD11 and PLCG1 activate NF-κB pathways, contributing to tumor growth (30). Other less frequent mutations involve genes like PDPK1, CTNNB1, MAPK, KRAS, and STAT3 (31–33).

### 3.4 Relationship between AITL and EB virus

The Epstein-Barr virus (EBV) is detectable in most AITL patients, primarily in B cells rather than tumor T cells. Its exact role in AITL development is unclear. Ebv-infected B cells transmit EBV protein signals to T cells through major histocompatibility complex (MHC) class II molecules, where they bind to T-cell receptors. This process provides antigen and costimulatory signals for T cell activation and promotes TFH self-proliferation. Some studies have used high-throughput sequencing to compare the EB virus transcriptome between patients with AITL and other lymphomas. They showed that the co-expression of EB virus-related genes in AITL may contribute to immune escape or survival of infected cells, thereby promoting AITL development (34) (35). Et al. showed that EBV virus was clonal and latent in all AITL samples. This suggests that EBV may be involved in the pathological process of AITL (36). Shi et al. found that a high count of EBV-positive cells (>50/HPF) was associated with a significantly worse prognosis and was an independent prognostic indicator for OS and PFS (37).

## 4 Treatment

Currently, the first-line treatment for AITL is still anthracycline-based chemotherapy. Most patients still show low remission rate, rapid progression and high recurrence rate after first-line treatment. Targeted therapy related to gene mutations provides a new strategy for the treatment of AITL and is expected to improve the prognosis of AITL patients.

### 4.1 First-line treatment

AITL remains anthracycline-based chemotherapy regimens, such as CHOP (cyclophosphamide, doxorubicin, vincristine, and prednisone). However, these regimens have demonstrated limited efficacy, with patients often experiencing low remission rates, rapid disease progression, and high relapse rates. To improve patient outcomes, researchers are exploring enhancements to first-line therapies, including the incorporation of additional agents into standard chemotherapy, combination therapies with novel drugs, and the use of ASCT as consolidation therapy. This section reviews the current approaches to first-line treatment for AITL, focusing on chemotherapy regimens, combination strategies, and the role of ASCT in improving long-term survival.

#### 4.1.1 Chemotherapy

Currently, the anthracycline-based CHOP (like) regimen (cyclophosphamide + doxorubicin + vincristine + prednisone) is the first-line treatment for AITL, but the five-year OS rate is only 32%–36% (38). A prospective cohort study in the United States compared various first-line treatment regimens for PTCL, including doxorubicin, etoposide, gemcitabine, and doxorubicin + etoposide, as well as other single-drug or combination regimens. It found that patients treated with doxorubicin had a longer survival time, so anthracyclines are still an important part of initial treatment. Improvements to first-line treatment methods include adding other potentially effective drugs to CHOP and first-line autologous hematopoietic stem cell transplantation (ASCT) consolidation therapy (39). Schmitz et al. (40) analyzed 343 patients with extranodal mature T-cell or natural killer cell lymphoma in the German Study Group for High-Grade Non-Hodgkin Lymphoma (DSHNHL) trial. They found that young patients with PTCL (<60 years old) with normal LDH levels who received CHOP combined with etoposide treatment had improved three-year event-free survival (EFS) by up to 75.4%, while OS was not significantly affected (**Table 1**).

**Table 1:** Summary of efficacy of drugs in AITL.

TREAMENT	Condition	Patient (n)	Efficacy			
			ORR (%)	CR(%)	PFS	OS
CHOP (38)	AITL	207	NA	NA	38%(3years)	54%(3years)
BV+CHOP (42)	AITL	30	NA	NA	26.6%(5years)	67.8%(5years)
Lenalidomide+ CHOP (44)	AITL	44	56.4%	41.0%	42.1%(2years)	59.2%(2years)
Chidamide (54)	R/R AITL	10	50.0%	40.0%	NA	21.4m
Chidamide+CHOP (56)	R/R AITL	33	81.8%	72.7%	22m	not reached
CSA (76)	R/R AITL	12	75%	33.30%	25.5m	NA
Tipifarnib (77)	R/R AITL	38	56.3%	28.1%	3.6m	32.8m
MEDI-570 (90)	R/R AITL	16	44.0%	12.5%	2.9m	17.1m

AITL, angioimmunoblastic T-cell lymphoma; R/R, relapsed/refractory; ORR, objective response rate; CR, complete response; PFS, pro9gress free survival; OS, overall survival; CHOP, cyclophosphamide, doxorubicin, vincristine, and prednisone; BV, brentuximab vedotin.

#### 4.1.2 Combination therapy

The US Food and Drug Administration (FDA) approved the combination of brentuximab vedotin (BV) and CHP (cyclophosphamide + doxorubicin + prednisone) to treat newly diagnosed CD30^+^ PTCL. Its approval was based on an open, multicenter, randomized phase III clinical trial, ECHELON-2, which compared the efficacy of BV+CHP to CHOP in 452 patients newly diagnosed with PTCL with ≥10% CD30^+^ cells. The results showed that the BV+CHP group had a median PFS of 48.2 months, with a 29% lower disease recurrence rate and 34% lower mortality rate than the control (CHOP) group. In patients with AITL, the estimated 5-year OS was 67.8% for A+CHP and 62.5% for CHOP (41, 42). *CD30* is expressed in 63%~75% of AITL tumor cells, providing a pathological basis for implementing this regimen (43). A multicenter prospective phase II study combined lenalidomide with CHOP to treat patients newly diagnosed with AITL aged 60–80 years. However, this regimen did not improve the complete metabolic response (CMR) rate. Further analysis showed that *DNMT3A* mutations were associated with reduced CMR rates and shortened PFS (44). Everolimus is a mammalian target of rapamycin (mTOR) inhibitor. Kim et al. (45) conducted a phase II study of combining everolimus with CHOP as a first-line treatment for patients with PTCL. The objective response rate (ORR) was 90%, and all three patients with AITL achieved a complete response (CR). However, the response duration of this regimen was relatively short, with a two-year PFS rate of only 33%. A prospective phase II clinical trial in China combining recombinant human endostatin (Endostar) with CHOP enrolled 15 patients with PTCL. All three enrolled patients with AITL achieved a CR, and the five-year OS rate was 100%. Two of the patients with AITL had elevated VEGF receptor 2 (*VEGFR2*) expression, suggesting that this regimen may benefit patients with AITL with *VEGFR2* overexpression, and further studies are needed to confirm this hypothesis (46).

#### 4.1.3 ASCT

Regarding consolidation therapy, Park et al. (47) directly compared the survival outcomes of patients with PTCL who received or did not receive ASCT consolidation therapy as first-line therapy. A CR was achieved by patients with PTCL who were sensitive to first-line therapy. The median follow-up of 2.8 years showed that for patients with AITL with a CR, the median PFS and OS were not reached in the ASCT group, while the median PFS and OS were 18.6 months and 24.3 months in the non-ASCT group, respectively. The Fukuoka Blood and Marrow Transplantation Group analyzed the clinical efficacy of stem cell transplantation (SCT) in treating patients with PTCL-NOS or AITL. For patients with AITL, the three-year OS rates for autologous or allogeneic SCT were 68.6% and 100%, respectively, while the three-year OS rate for non-SCT was 57.2% (*P* = 0.018) (48). A prospective randomized trial by the German High-Risk Lymphoma Collaborative Group showed that the three-year EFS and OS rates were comparable between patients with PTCL who received ASCT or allogeneic hematopoietic SCT (allo-SCT) as first-line consolidation therapy (49). Therefore, for young patients with PTCL who are eligible for transplantation and are sensitive to chemotherapy, ASCT can be given priority as first-line consolidation therapy.

### 4.2 Relapse and refractory disease

Advances in understanding the genetic and molecular mechanisms of AITL have led to the development of targeted therapies aimed at specific pathways involved in disease pathogenesis. This section discusses current strategies for treating relapsed and refractory AITL, including the use of histone deacetylase inhibitors, DNA methyltransferase inhibitors, monoclonal antibodies targeting CD30, PI3K/AKT pathway inhibitors, and other emerging therapeutic options that offer hope for improved patient outcomes.

#### 4.2.1 Histone deacetylase inhibitors

**Figure 1:**
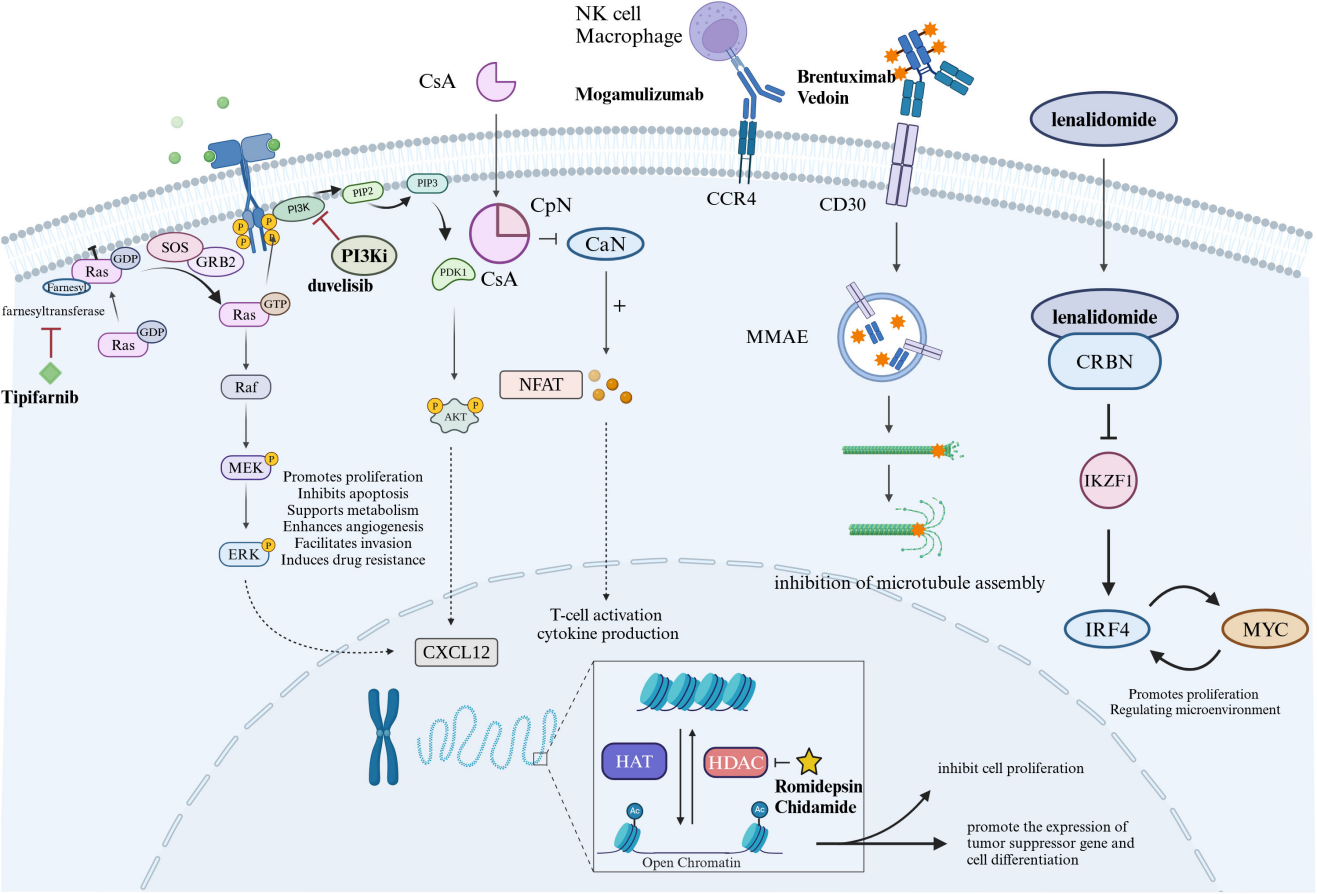
Diagram of drug mechanism of action in the treatment of AITL. Tipifarnib is a selective farnesyl transferase inhibitor that targets RAS proteins. Duvelisib is an oral PI3K inhibitor. Cyclosporin A has immunosuppressive effect on T lymphocytes. Mogamulizumab is a monoclonal antibody against C-C motif chemokine receptor 4 (CCR4) that kills tumor cells through antiboy-dependent cell-mediated cytotoxicity. BV is an anti-CD30 antibody-drug conjugate that acts on CD30 targets on tumor cells to kill tumor cells. Lenalidomide produces anti-tumor effects through immune regulation and inhibition of downstream IRF4, MYC, etc. Histone deacetylase (HDAC) is involved in chromatin remodeling, and HDAC inhibitors (HDACi) have antitumor effects.

Patients with AITL have high histone deacetylase (HDAC) activity, which is involved in chromatin remodeling and is important in the epigenetic regulation of gene expression as shown in **Figure 1**. In clinical studies, HDAC inhibitors (HDACi) have shown potential anti-tumor activity and have pleiotropic effects, including gene regulation, cell cycle arrest, anti-angiogenesis, and activation of apoptosis (50). Current HDACi include romidepsin, belinostat, vorinostat, and chidamide. Similarly, HDACi also has active effects in AITL, which may be related to the high frequency of mutations in epigenetic modifier genes that occur in this disease.

A single-arm, phase 2 prospective study used romidepsin, an HDACi, as monotherapy for patients with relapsed/refractory (R/R) PTCL. It included 131 patients (27 with AITL), achieving an ORR of 25% and a median duration of remission of 28 months. The longest duration of remission in patients with AITL was 56 months (51). Another study (52) that treated seven cases of R/R AITL with romidepsin combined with lenalidomide or lenalidomide and carfilzomib found an ORR of 87% and CR of 57%, which were significantly higher than those in the control group. However, some studies have shown that combining romidepsin with chemotherapy does not prolong the PFS of patients with PTCL compared to chemotherapy alone (53), so whether it can be used in combination remains to be further confirmed. Chidamide is a novel oral selective HDAC inhibitor. A multicenter phase II clinical trial in China showed that the median OS of patients with R/RAITL treated with chidamide was 21.4 months, significantly higher than the 11.3 months of romidepsin (54). Another clinical study used the HDACi chidamide to treat patients with PTCL, achieving a total ORR of 46%, with five cases achieving a CR. Subgroup analysis showed that among the eight patients with AITL, two achieved a CR, five achieved a partial response (PR), and the ORR was 88% (55), indicating that cedabenamide has a good effect in treating AITL. One study evaluated the efficacy of chidamide in combination with CHOP in patients newly diagnosed with AITL. Median overall survival and median progression-free survival were significantly longer in the chidamide combined chemotherapy group compared with the control group’s CHOP regimen (56). Ghione et al. (57) retrospectively analyzed the efficacy of HDACi monotherapy or combination therapy in 127 patients with R/R PTCL in six centers. Patients with TFH phenotype lymphoma achieved better efficacy, with ORRs of 54.2% and 61.1% for monotherapy and combination therapy, respectively, and had a slightly longer PFS. Moreover, 18% could use HDACi as a bridge to allogeneic transplantation.

#### 4.2.2 DNA methyltransferase inhibitors

Hypermethylation of the promoter regions of tumor suppressor and DNA repair genes can downregulate their expression, resulting in disordered differentiation of normal cells and failure to repair DNA damage, which can lead to tumors. Currently, increasing clinical trials are focusing on azacitidine or other DNA methyltransferase inhibitors (58). Lemonnier et al. (59) retrospectively analyzed the efficacy of an azacitidine-containing regimen in 12 patients with AITL, achieving an ORR of 75% and CR rate of 41%. Subsequently, O’Connor et al. (60) conducted a multicenter phase I clinical trial of azacitidine combined with romidepsin to treat R/R lymphoma. Eight out of 11 patients with T cell lymphoma (TCL) responded, and three patients with AITL achieved a CR. Yoon et al. retrospectively analyzed the efficacy and safety of azacitidine as a salvage chemotherapy regimen in 15 patients with R/R AITL and found that patients with less than 2 previous chemotherapy had a higher ORR than those with more than 2 previous chemotherapy, and patients who received the full dose showed a better ORR than those who did not receive the optimal dose (61). Another phase II trial proved that for patients with PTCL with a TFH phenotype, dual epigenetic inhibitor combination therapy has a higher response rate and longer response time, with an ORR of 80%, a CR rate of 60%, and a median PFS of 8.9 months (62). A recent multicenter phase II clinical study of CHOP combined with azacitidine for the treatment of newly diagnosed PTCL showed that in patients with TFH phenotype, the CR rate can reach 88%, and the two-year OS is 76%, and patients with TET2 mutations have higher CR, PFS and OS (63).

#### 4.2.3 CD30 monoclonal antibodies

BV is an anti-CD30 antibody-drug conjugate that can kill tumor cells by directly acting on the CD30 target on tumor cells. It was approved in 2018 to treat patients with CD30^+^ PTCL undergoing initial treatment. Horwitz et al. (64) evaluated the efficacy and safety of BV in treating R/R CD30^+^ non-Hodgkin’s lymphoma (NHL). The ORR was 54% (five CRs, two PRs), and the median PFS was 6.7 months in the AITL group. Oberic et al. (65) retrospectively analyzed the efficacy of BV alone or in combination with chemotherapy in treating patients with R/R CD30^+^ TCL. The CR rate was 40.7%, and the median PFS and OS were 5.2 and 12.5 months, respectively. Therefore, BV can serve as a bridge to transplantation for young patients with R/R TCL who are sensitive to chemotherapy. A study of 82 patients confirmed that the combination of bendamustine and BV showed promising results in R/R PTCL. The optimal overall response rate (ORR) was 68%, with 49% of patients achieving complete response (CR). The median duration of response for CR patients was 15.4 months, median follow-up was 22 months, and median progression-free survival (PFS) and overall survival (OS) were 8.3 and 26.3 months, respectively (66).

#### 4.2.4 PI3K/AKT pathway inhibitors

PI3K/AKT pathway inhibitors are active and safe in patients with PTCL. Duvelisib is an oral PI3K-δ/γ isoform inhibitor. One study (67) showed that duvelisib has some efficacy in treating different types of R/R lymphomas. The overall ORR was higher for patients with AITL than those with other PTCL. The incidences of any grade, grade ≥3, serious adverse reactions, treatment-related discontinuation, and death were 99%, 79%, 63%, 33%, and 3%, respectively. The treatment risk and efficacy of duvelisib may be further reduced through proper identification and management. Horwitz et al. (68) conducted a duvelisib dose escalation trial in 35 patients with R/R TCL. The ORR was 50.0% in the PTCL group; of the three patients with AITL, one achieved a CR, and one achieved a PR. A multicenter phase I/II clinical trial using copanlisib combined with gemcitabine to treat R/R PTCL found that the CR rate of the AITL subtype was 55.6%, and sustained remission was achieved (69). The JAK/STAT signaling pathway also exists in AITL. A multicenter phase I clinical trial using the JAK1/2 inhibitor ruxolitinib to treat newly diagnosed T-cell lymphoma included 9 AITL patients. The ORR was 33%, but only 1 patient achieved CR. The results suggest that although ruxolitinib is effective for AITL patients, it is not suitable for single-agent use (70).

#### 4.2.5 Others

Lenadomide is an immunomodulator that can produce anti-tumor effects directly or indirectly through its unique immune regulation. Hu et al. (71) treated a patient with diffuse large B cell lymphoma combined with AITL with lenalidomide. A CR was achieved after six courses of the lenalidomide + R-miniCHOP regimen. Continued lenalidomide monotherapy as maintenance therapy achieved sustained remission. The hematological toxicity was mild during treatment, and the patient’s tolerance and compliance were good. In addition, the broad-spectrum multikinase inhibitor dasatinib can effectively inhibit the abnormal activation of VAV1 and subsequent TCR signal transduction caused by the RHOA G17V and VAV1 mutations in AITL. Therefore, it could be used as a new treatment strategy for AITL (72). Mogamulizumab is a monoclonal antibody against C-C motif chemokine receptor 4 (CCR4) and can be used as an important supplement to TCL drug treatment. It acts through antiboy-dependent cytotoxic activity, reducing regulatory T cells and stimulating anti-tumor effects. Results from a multicenter Phase 2 clinical trial confirmed 50% ORR for mogamulizumab in the treatment of AITL (73). A retrospective study of 39 patients with AITL confirmed that patients treated with chemotherapy combined with mogamulizumab had a four-year overall survival rate of 46.3%, compared with 20.6% in the chemotherapy alone group (74). Mogamulizumab is also approved by the European Medicines Agency (EMA) for the treatment of relapsed or refractory peripheral T-cell lymphoma (PTCL). Cytotoxic T cells activated by EB virus-specific antigen peptides showed good efficacy and high clinical safety in treating R/R AITL (75). Immunosuppressive cyclosporin A also has certain potential in the treatment of AITL (76). Tipifarnib is a selective farnesyl transferase inhibitor that regulates C-X-C motif chemokine ligand 12 (CXCL12) expression. Tipifarnib monotherapy showed good efficacy in patients with R/R AITL (77). More targeted drugs are currently under clinical research (**Table 2**).

**Table 2:** Summary of ongoing clinical trials of AITL.

Experimental drug	Development stage	Condition	Status	NCT number
ARV-393	Phase I	R/R AITL	Recruiting	NCT06393738
ONO-4685	Phase I	R/R TCL	Recruiting	NCT05079282
ST-001	Phase I	R/R AITL	Recruiting	NCT04234048
BMS-986369	Phase IB/II	R/R TCL	Recruiting	NCT06035497
GNC-038	Phase IB/II	R/R AITL	Recruiting	NCT05627856
Mitoxantrone Liposome+ Azacitidine	Phase IB/II	R/R AITL	Recruiting	NCT06224842
Lenalidomide + CHOP	Phase II	AITL	Recruiting	NCT04423926
Soquelitinib	Phase III	R/R AITL	Recruiting	NCT06561048
Ro Plus CHOEP	Phase I/II	AITL	Active, not recruiting	NCT02223208

AITL, angioimmunoblastic T-cell lymphoma; TCL, T-cell-leukemia-lymphoma; R/R, relapsed/refractory; CHOEP, etoposide, cyclophosphamide, doxorubicin, vincristine, and prednisone.

## 5 Clinical prognostic factors

The International Prognostic Index (IPI), commonly used for non-Hodgkin lymphoma (NHL), is unsuitable for AITL as it does not significantly correlate with overall survival (OS) (78). CRP is an inflammatory marker important in lymphoma prognosis. A retrospective analysis of 52 AITL patients found that high serum CRP levels (>20 mg/L) are closely associated with poor OS (30). IRF4 is a biomarker and potential therapeutic target in peripheral T-cell lymphoma due to its expression in T-cell lymphomas (79, 80). Its expression correlates with poor prognosis in AITL; patients with high IRF4 levels have worse survival rates (81). Lenalidomide, targeting IRF4 and MYC, has shown efficacy in AITL, suggesting combination therapies may improve outcomes (82). IL-10 induces an immunosuppressive environment, hindering anti-tumor responses. In AITL, high serum IL-10 levels are linked to lower complete response rates to CHOP chemotherapy and decreased OS (83), making it a potential specific prognostic marker. ctDNA contains tumor-specific mutations and serves as a biomarker for monitoring treatment efficacy. In AITL, ctDNA effectively detects RHOA G17V mutations with high sensitivity and specificity (84, 85). Persistent ctDNA post-treatment indicates poor prognosis, highlighting its value in non-invasive monitoring (86). Studies in other cancers, such as colorectal and hepatocellular carcinoma, demonstrate the prognostic significance of ctDNA, suggesting its potential applicability in AITL (87). VEGF promotes angiogenesis and is overexpressed in AITL, correlating with poorer disease-free survival and OS (88). Anti-angiogenic therapy with agents like recombinant human endostatin (Endostar) combined with chemotherapy has shown potential benefits in AITL patients, improving response rates and survival (89).

## 6 Future perspectives

Genetic analysis plays an important role in the management of AITL. The gene expression profile of AITL can provide a more accurate diagnosis and classification than immunohistochemistry, which helps distinguish AITL from other types of peripheral T-cell lymphoma (10). In addition, gene mutations are associated with the occurrence, development and prognosis of AITL. Genetic analysis can provide the basis for personalized treatment. Therefore, genetic analysis is recommended in the future management of AITL. At present, CHOP, BV-CHP, lenalidomide and autologous hematopoietic stem cell transplantation are still recommended for the treatment of AITL. PI3K inhibitors such as duvelisib may be considered for patients with altered PI3K pathways. For patients with epigenetic alterations, HDAC inhibitors may be considered as a potential treatment option. A number of targeted therapies and immunotherapy strategies are under investigation, including those targeting PD-1, ICOS (90), CD30 and others. CAR-T cell therapy targeting specific surface markers of AITL, such as CD4, is under investigation (8). We look forward to providing more precise treatments in the future.

## 7 Conclusion

AITL is a specific type of PTCL that is highly aggressive. It is common in older patients and has complex clinical manifestations. Its prognosis is worse than other PTCL types, and its treatment is challenging. In addition, AITL is a type of PTCL with unique biological behavior, and its onset may be related to infection, cytogenetic changes, and immune abnormalities. Its current first-line treatment regimen is similar to other PTCL types and is still based on the CHOP (like) regimen. Patients effectively treated by first-line therapy can consider first-line ASCT for consolidation. New drug combination regimens based on this are being explored. For R/R patients, ongoing studies are evaluating new potential targets for AITL, including new targeted drugs such as rituximab, epigenetic drugs, and PI3K inhibitors, which have shown significant clinical efficacy in AITL. In the future, these may become an important choice for patients with relapsed, refractory, or even newly diagnosed AITL. As in-depth research on its pathogenesis progresses, more and more new drugs and treatments have been discovered and applied clinically. However, there remains no unified standard for treating AITL, and further exploration is needed to identify a satisfactory treatment plan. Further research is required to obtain satisfactory efficacy.
